# Achievement in Fundamental Movement Skills, Spatial Abilities, and Mathematics among Lower Key Stage 2 Children

**DOI:** 10.3390/jintelligence11050087

**Published:** 2023-05-04

**Authors:** Jessica Scott, Tim Jay, Christopher Spray

**Affiliations:** 1 School of Sport, Exercise and Health Sciences, Loughborough University, Loughborough LE11 3TU, UK; 2 Department of Mathematics Education, Loughborough University, Loughborough LE11 3TU, UK

**Keywords:** fundamental movement skills, FMS, spatial ability, mathematics achievement

## Abstract

Research has demonstrated links between sport and mathematics learning, and their relationship with spatial abilities in children. This study explored the association between the development of fundamental movement skills (FMS) and mathematics achievement, and whether the understanding of specific spatial concepts mediated these relationships. Overall, 154 Year 3 children (69 males, 85 females, aged 7–8 years) from four schools in England completed an FMS assessment involving six skills; four spatial tasks assessing intrinsic-static, intrinsic-dynamic, extrinsic-static, and extrinsic-dynamic spatial abilities; and a mathematics test assessing numerical, geometrical, and arithmetical abilities. Overall FMS ability (a combined score across the six skills) was significantly positively correlated to overall mathematics achievement. This relationship was mediated by children’s performance on the intrinsic-static spatial ability test. These findings suggest that children who have more mature FMS perform better in mathematics tasks, and this could be due to more developed intrinsic-static spatial ability. However, further research is necessary to determine the mediation effects of intrinsic-dynamic and extrinsic-static spatial ability.

## 1. Introduction

Fundamental movement skills (FMS) are basic locomotor, object manipulation, and stability patterns that lead to complex specialised skills (Gallahue and Ozmun 2006; Rudd et al. 2015). They contribute to the holistic development and well-being of children (Brown and Cairney 2020). Duncan et al. (2018) report that underdeveloped FMS in children may hinder the development of a physically active lifestyle as they grow older and result in long term well-being problems (Barnett et al. 2016). The beneficial effects of mature FMS and thus physical activity on one’s physical and mental health and well-being have been thoroughly examined (Bize et al. 2007; Lee et al. 2012). However, what is less well known about FMS is their relationship with academic performance, specifically in mathematics. The main goal of this study is to investigate the relationships between FMS performance, achievement in mathematics, and spatial abilities in primary school children.

In the UK, by the end of Key Stage 1 (8-years-old), children should be able to master FMS (Department for Education 2013), which are taught and developed throughout their PE lessons (Bailey 2006). Locomotor movements involve the body moving from one place to another in any direction, for example, running, hopping, or jumping (Gallahue and Ozmun 2006). The object manipulation construct is formed of movements that involve giving or receiving force to or from an object using a body part or tool, such as catching, throwing, kicking, and striking (Gallahue and Ozmun 2006). Stabilisation, which Rudd et al. (2015) propose is as important as locomotion and object manipulation skills, are movements that involve the body remaining in place whilst either being stationary or moving around its horizontal and vertical axis, for example, turning, twisting, and static and dynamic balances (Gallahue and Ozmun 2006).

Mathematics is a valued, core, and compulsory academic subject globally (Maass et al. 2019). It is important for everyday life (Organisation for Economic Co-operation and Development 1999) and benefits nations economically (National Mathematics Advisory Panel 2008); therefore, mathematics skills in children are considered important. Mathematical achievement in early childhood is related to long term academic success (Duncan et al. 2007) and poor achievement in mathematics has been associated with underperformance in general academic achievement and poor future economic and health outcomes (Butterworth et al. 2011). However, many school children do not like mathematics and consider it a difficult and demanding subject, with many wishing they no longer had to study the subject (Haag and Gotz 2012, as cited in Heyder et al. 2020; Sharma 2008). Students have reported that they are more anxious about mathematics than other academic subjects (Goetz et al. 2007) and mathematical anxiety is evident in young children (Ramirez et al. 2013). This may be a reason why internationally there are declines in mathematical achievement among students (Wijsman et al. 2016). Within the UK, the Department for Education (2015) has discovered that many children do not meet the required levels of mathematics needed by the end of primary school to be ready for secondary school. Therefore, if we can identify the factors that may positively affect mathematics performance early on, more benefits will hopefully occur later in life.

FMS are associated with mathematical achievement. Overall FMS scores have been found to be positively associated with mathematics scores in early childhood (Willoughby et al. 2021), primary school (de Bruijn et al. 2019), and secondary school-aged children (Ericsson and Karlsson 2014; Jaakkola et al. 2015). Very few studies have looked at the specific relationships between different FMS and mathematical abilities, such as numerical, arithmetical, geometrical, and logical reasoning, as defined by Xie et al. (2020). In a study conducted by de Waal (2019), different FMS within the locomotion, stability, and object manipulation constructs were investigated in relation to numerical and geometrical mathematical skills in 5-to-6-year-old children. de Waal (2019) found that hopping, jumping, gliding, skipping, and dynamic and static balancing were positively correlated with numerical ability, and all but skipping correlated positively with geometrical ability. No FMS correlated with measurement. Further, there were no correlations between any of the object manipulation skills (catching and overarm throw) and mathematical abilities. These results highlight that there may be specific relationships between certain FMS and mathematical abilities, which requires further examination to better understand the mechanisms or mediators of the relationship between FMS and mathematics.

**Figure 1:**
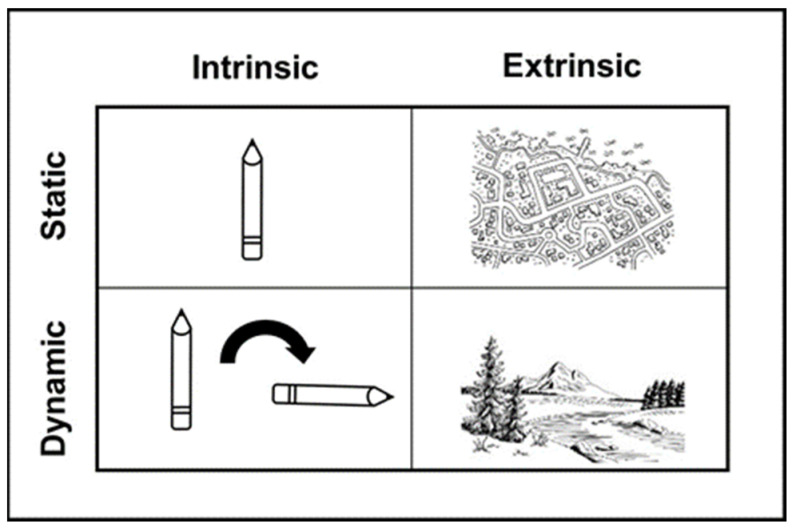
Uttal et al.’s (2013) 2 × 2 classification of spatial abilities (adapted from Uttal et al. (2013)).

There is a need to explain why an association between FMS and achievement in mathematics has been found; spatial ability may be an intermediary factor to explain this association. Spatial ability is defined as the capacity to process, memorise, recall, and transform visual stimuli to comprehend visual-spatial relationships (Linn and Petersen 1985). Uttal et al.’s (2013) approach classifies spatial skills by distinguishing between intrinsic and extrinsic (defining the spatial relationships within objects or between objects in a group relative to one another), and static and dynamic spatial skills (defining spatial characteristics without or with the need for mental or physical transformation). This results in the creation of a 2 × 2 classification of spatial ability: intrinsic-static (IS); intrinsic-dynamic (ID); extrinsic-static (ES); and extrinsic-dynamic (ED), see Figure 1. Despite factor analysis research not confirming Uttal’s distinction of four spatial skills via a four-factor model in children (Mix et al. 2018), which may be due to limited and complicated research into the distinction between static and dynamic skills in children (Newcombe 2018), Uttal’s model is used in this study to assess spatial skills. This selection is based on the evidence that spatial ability is not a unitary construct (Newcombe and Shipley 2015). Behavioural evidence in young children supports the distinction between intrinsic and extrinsic skills (Mix et al. 2018), and behavioural evidence in adults supports the static-dynamic dimension (Kozhevnikov et al. 2005). Further, Gilligan et al. (2019) found that the model’s four spatial skills had varying roles in explaining academic achievement, specifically in mathematics, supporting the distinction between both the intrinsic-extrinsic and static-dynamic dimensions in children.

Regarding the relationship between FMS and spatial ability, adults who participate in sports have better spatial skills than those who do not (Moè et al. 2018). Further, Moraweitz and Muehlbauer (2021)’s review found that spatial abilities could be improved through physical activity interventions in children and adolescents, highlighting that physical activity and spatial abilities are positively related and may positively influence one another across all ages. When examining the associations with specific spatial skills, most of the research focuses on ID spatial ability. Girls aged between 6 and 14 years old had improved mental rotation performance after completing a three-month juggling programme that incorporated high coordination demands than girls who completed a light strengthening programme (Jansen et al. 2011), and mental rotation performance in first and second graders was improved in children who attended a dance training group compared to a traditional PE lesson (Jansen and Richter 2015). This highlights that spatial ability, specifically mental rotation ability, is positively related to physical activity in children.

Potential mechanisms that may explain this relationship between motor and spatial abilities, specifically ID spatial ability, have been proposed. For example, activation in motor areas of the brain have been found during mental rotation tasks (Kosslyn et al. 2001). In addition, mental rotation reaction times were slower when children performed a motor task simultaneously, highlighting a motor interference on mental rotation (Frick et al. 2009). Therefore, it appears that mental representations and transformations are connected to sensory and motor systems, which could explain the observed relationship found between physical activity, motor skills, and spatial ability.

Evidence shows consistent positive associations between mathematical ability and spatial ability (Atit et al. 2022; Frick 2019), and this relationship is evident in infancy and continues through to adulthood (Verdine et al. 2017). Research has found a link between IS spatial skills and numerical and arithmetical ability in young children (Harvey and Miller 2017; LeFevre et al. 2013). There is also evidence supporting a positive relationship between ID spatial ability and numerical (Cornu et al. 2018; Fernandez-Mendez et al. 2021; Hawes et al. 2015), arithmetical (Zhang et al. 2017; Zhou et al. 2015), and logical reasoning ability (Boonen et al. 2014; Oostermeijer et al. 2014) in 5-to-10-year-old children. ES spatial ability has also been found to be positively related with numerical (Anobile et al. 2018), arithmetical, and logical reasoning ability (Zhang and Lin 2015) in children and geometrical ability in adolescents (Goldsmith et al. 2016). Recent research in spatial training has found significant improvements in mathematics after participating in spatial training compared to control groups in children and young adults, with the effects increasing with age (Hawes et al. 2022). This convincingly highlights that spatial skills may exert a causal effect on mathematics learning.

These studies support the conclusions made by Xie et al.’s (2020) meta-analysis of 73 studies, which found that IS, ID, and ES were significantly positively related to arithmetic, numeracy, geometry, and logical reasoning. ED spatial ability was not associated with mathematics. However, the conclusion that ED spatial ability is not related to mathematics should be viewed cautiously as few effect sizes were reported for ED spatial ability. This conclusion also contrasts the results found by research in children which used perspective taking tasks to assess ED spatial ability (Gilligan et al. 2019; Mix et al. 2016; Tam et al. 2019). This difference could be due to Xie et al.’s (2020) meta-analysis comprising an overview of studies with children, adolescents, and adults, and various methods to assess each spatial ability were used. Therefore, when the most appropriate tests are used for the age group being assessed, it appears that there is a significant positive relationship between all spatial ability skills and all areas of mathematical ability.

Theoretical explanations for why spatial skills and mathematics may be related have been proposed. The first is that numbers are spatially represented along a mental number line, with smaller number magnitudes being represented on the left and larger number magnitudes represented on the right (Gunderson et al. 2012). In addition, evidence highlights that spatial skills and mathematics skills share underlying neurology. For example, the parietal and frontal cortex are activated in both spatial and mathematics tasks (Hawes et al. 2019). Another explanation may be that mathematical skills are linked to embodied cognition (Lakoff and Nunez 2000). This theory proposes that perceptual spatial skills aid individuals to solve mathematical problems because mathematical concepts become more meaningful when grounded with daily sensorimotor and visual-perceptual experiences. This evidence further adds to the literature to support relationships between mathematics and spatial abilities.

One study has investigated specific FMS in relation to specific spatial abilities and mathematical abilities. Jansen and Pietsch (2022) investigated the relationships between running, jumping, and throwing, and their equivalent spatial ability dimension, as per Pietsch (2018)’s visual-spatial taxonomy of sport skills, and mathematics performance in 8-to-10-year-old children. There were positive relationships between running and IS spatial ability, as assessed by the visual figures test, and throwing and ES spatial ability, as assessed by the water level test, but no relationship was found between jumping and ID spatial ability, as assessed by mental rotation. Performance in arithmetical, numerical, and geometrical ability was assessed and the only significant positive correlation found was between IS spatial ability and geometrical ability, specifically in boys; the more items solved in the visual figures test, the better the children’s geometrical scores. However, geometrical ability could not be predicted by sprinting ability; performance in sprinting only explained 7% of the variance in geometrical ability. This is the first study to investigate the potential relationship between achievement in FMS and mathematics by focusing on specific abilities and incorporating spatial ability into the relationship. As some of the FMS assessed were not age-appropriate, ED spatial ability was omitted and not all areas of mathematical ability were assessed, further research into this area is necessary to highlight the potential beneficial impact FMS may have on achievement in mathematics.

To address the need for further research in this area, to help clarify the relationships between FMS, spatial skills, and mathematics achievement, the present study hypothesised that total FMS score is positively associated with higher performance in mathematics (H1) and that spatial ability will mediate the relationship between FMS and mathematical achievement (H2). Previous research has only focused on locomotion and object manipulation skills, omitting stability skills. In addition, ED spatial ability has previously been assessed with age-inappropriate tests, which has led to inconclusive conclusions being determined between mathematics and spatial ability. Therefore, the current study aims to address these limitations by combining FMS performance, spatial ability, and mathematical ability in one study. All three constructs of FMS will be assessed, as well as all four spatial abilities as according to the 2 × 2 classification described by Uttal et al. (2013), using age-appropriate tests. To assess mathematics achievement, a UK standardised test covering several areas of numeracy, arithmetical ability, and geometry, as described by Xie et al. (2020), will be administered.

## 2. Materials and Methods

### 2.1. Participants and Procedure

Four primary schools consisting of 178 Year 3 children from the UK were approached. Approval for the research to take part within the school was granted by the headteachers. A favourable ethical decision was also granted by the Ethics Review Sub-Committee at Loughborough University. Parents and guardians were sent information sheets and parental consent forms via the school channels. Children who had been given informed parental consent and who gave their own informed assent participated in the study.

Overall, 155 Year 3 children participated in the study (65 boys, mean age: 8.04, SD = .301). The ethnicity of the sample was mostly White British (75.5%, *n* = 117). The sample represented these other ethnic groups: White and Black Caribbean (3.2%, *n* = 5); White and Asian (2.6%, *n* = 4); White and Black African (2.6%, *n* = 4); Pakistani (1.9%, *n* = 3); Bangladeshi (1.9%, *n* = 3); Any Other Asian Background (1.9%, *n* = 3); White Other (1.3%, *n* = 2); Indian (1.3%, *n* = 2); Chinese (1.3%, *n* = 2); Black Caribbean (1.3%, *n* = 2); Other (1.3%, *n* = 2); Any Other Mixed Background (1.3%, *n* = 2); and Black African (0.7%, *n* = 1). The ethnicities of three children were not disclosed. In addition, 17 children (35.3% boys) received financial school aid, 21 children (57.1% boys) had a learning disability, and 4 children (50% boys) had a physical impairment. The sample size meets the required sample size needed for a mediation analysis. According to Fritz and MacKinnon (2007), when conducting a simple mediation analysis using the bootstrapping (bias-corrected) method, for an anticipated effect size of .26 for both paths a and b, a statistical power of 80%, and a probability level of .05, a minimum of 148 participants is needed.

Data collection was conducted via a cross-sectional correlational design during the second half of the school year (March to June 2022). FMS, spatial ability, and mathematics ability were assessed on different days. FMS were assessed first during a PE lesson. Children watched the researcher demonstrate the first skill, then five children at one time performed this skill and were assessed by the researcher. Once all the children had been assessed on this skill, the researcher demonstrated the second skill and this method repeated until all skills were assessed. This took approximately one hour to complete for 30 children in a class. The following week, spatial abilities were assessed. Each child was assessed independently and completed the four tests on paper. This took approximately 30 min to complete per child as no time limits were enforced. Mathematics ability was assessed the following week during a mathematics lesson. Each child completed their own paper, and this took approximately 45 min to complete.

### 2.2. Measures

#### 2.2.1. FMS

**Figure 2:**
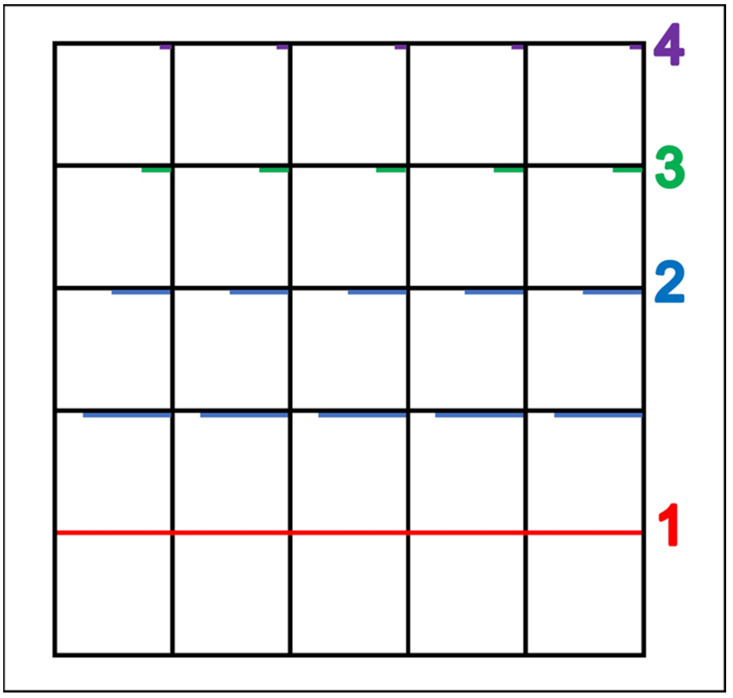
Grid layout to assess the FMS of five children at one time using FUNMOVES. The first target line is one meter long and this forms zone 1. The second target line is 75 cm long and the third target line is 50 cm long, both of which form zone 2. The fourth target line is 25 cm long and this forms zone 3, and the final target line is 10 cm long, forming zone 4.

FMS were assessed using the class-level product measure FUNMOVES (Eddy et al. 2021). FUNMOVES assesses locomotion (running, jumping, and hopping), object manipulation (throwing and kicking), and stability (static balances) skills. Each child is assessed along a one-metre by five-metre lane, with markings at every one-metre point, creating five one-metre by one-metre boxes. On these lines, there is an increasingly smaller target zone, marked in a different colour (see Figure 2).

Running is assessed by how many full lengths of the grid are run in 15 s. The jumping activity requires children to do as many small jumps as necessary to reach and stop still with both feet on the next target line, which becomes increasingly smaller. The assessor then counts for three seconds, and the children are then set off again to the next line. This is repeated until the children reach the back of the grid. Jumping is assessed on a scale of 1 to 4: 1—fails in zone 1, 2—can do the activity to halfway line (fails in zone 2), 3—can do the activity past halfway but cannot finish it (fails in zone 3), and 4—can complete the activity (does not fail). Falling, losing balance, not stopping on the line, or one or both feet is not on the coloured target line counts as failure. For the hopping task, children must perform as many hops as necessary on their chosen leg to reach and stop still on the next target line on one leg, landing on the increasingly smaller target lines, and wait until the assessor sets them off again. This is repeated until the children reach the back of the grid. This skill is also marked on a scale from 1 to 4, based on the zone that the children lose balance in, fail to land their foot on the coloured target line, put their other foot down on the ground, or shuffle whilst waiting on the coloured target line. In the present study, the three component scores for running, jumping, and hopping were combined to create a total locomotion score.

For the throwing skill, children must throw underarm one beanbag in each of the five one-metre by one-metre boxes in their lane. This is completed once for each arm. The kicking skill requires children to kick a beanbag along the floor into each of the boxes along their lane. This must only be done by the same leg, and only for one leg, chosen by the child. Both these skills are scored on a scale from 0 to 5, based on the number of boxes filled with a beanbag. In the present study the component scores for throwing and kicking were combined to create a total object manipulation score.

For the final skill, children must hold four balance positions: (i) balancing on both feet together with their eyes open and pass a beanbag around their body three times, (ii) balancing on one leg (chosen by the child) and pass a beanbag around their body three times, (iii) picking up a beanbag from the floor whilst balancing on one leg (chosen by the child), and (iv) balancing on one leg (chosen by the child) with their eyes closed and pass a beanbag around their body three times. This skill is scored on a scale from 0 to 4, based on how many balances were successfully completed. Once a balance is not completed correctly scoring is stopped for that child.

#### 2.2.2. Spatial Ability

**Figure 3:**
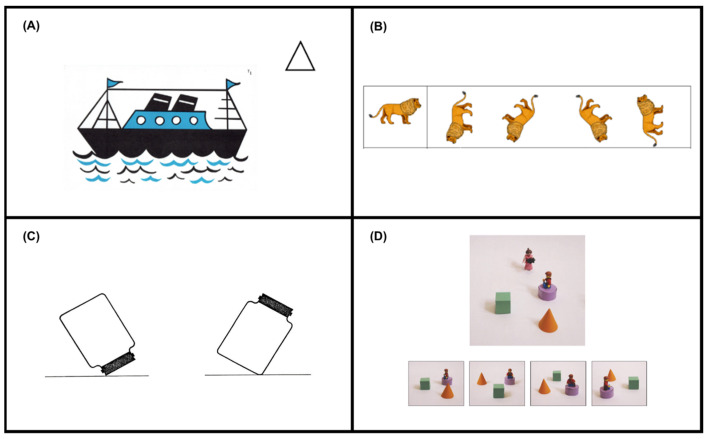
Example materials from the spatial tasks used in this research: (**A**) Children’s Embedded Figures Task, (**B**) Mental Rotation Task, (**C**) Water Level Task, (**D**) Perspective Taking Task.

IS Spatial Ability. The Children’s Embedded Figures Task (CEFT; Witkin et al. 1971) was used to assess IS spatial ability. Children are required to locate a target shape (a tent in block A and a house in block B) embedded within a more complex picture (see Figure 3 for an example). First, children complete discrimination, demonstration, and practice trails before the experimental trails (11 trials for block A, and 14 trials for block B). If children fail to identify the tent shape in all the 11 trials, they do not move onto block B. If a child does not correctly identify the house shape in five consecutive trials, the test is stopped. One point is awarded for each trial that has the shape correctly identified, resulting in a total score of 25. This test is reliable to use on 6-to-10-year-old children (Gilligan et al. 2019), with internal consistency coefficients ranging from .83 to .90 (Witkin et al. 1971).

ID Spatial Ability. The Picture Mental Rotation Task, animal stimuli (Neuburger et al. 2011), assessed ID spatial ability. Children must select the two pictures from the four on the right-hand side of the page that match the target item presented on the left-hand side of the page (see Figure 3 for an example). The two correct items are rotated versions of the target item; the other two are mirror images that have been rotated. The items are rotated at 45°, 90°, 135°, or 225° compared to the target item. The children are shown two examples and complete two practice trials before completing 16 experimental trials. One point is awarded if both correct pictures are crossed out, resulting in a total score of 16. Prior research has indicated good internal consistency (Cronbach’s alpha = .92).

ES Spatial Ability. The Water Level Test (Piaget and Inhelder 1956), as described in Jansen and Pietsch’s (2022) study, was used to assess ES spatial ability. Children are shown an image of a transparent jar half-full of water. The children must then draw the water surface line in 16 containers. Eight of the containers are identical to the example container but tilted in different positions; the other eight containers are of different designs and various sizes tilted at different angles (see Figure 3 for an example). One point was awarded for each correctly drawn horizontal line within a five-degree range either side of 180 degrees, resulting in a total score of 16.

ED Spatial Ability. The Perspective Taking Task (Frick 2019) was used to assess ED spatial ability. Children are required to identify the correct photograph that represents the picture taken by a character in a certain position based on a pictorial representation of the photographer in an arrangement. One of the four pictures shows the correct view, the other three are foils (see Figure 3 for an example). Children complete four introduction trials with real 3D objects and Playmobil characters, before completing five practice trails and 22 experimental trials. Complexity is introduced by increasing the number of objects in the stimulus picture (one, two, or four objects) and the objects differ in angular difference between the child and the photographer (0°, 90°, and 180°). A total score of 22 can be achieved. Previous research has highlighted that this test is reliable to use on this age group (Guttman’s Lambda-2 = .70, Frick 2019).

#### 2.2.3. Mathematics Ability

Mathematics ability was assessed using the class-level Mathematics Assessment for Learning and Teaching (MaLT; Williams 2005). For the sample age group, it was most appropriate to use the MaLT8, which is used for Year 3 children and has norms for ages 7 to 9.5 years. This assessment assesses areas of mathematics that match to the strands and learning objectives of the Primary Education Curriculum in England. These areas are counting and understanding number (CN, numerical ability), knowing and understanding number facts (NF, numerical ability), calculation (arithmetical ability), and understanding shape, measurement, and handling data, which are all geometrical abilities. The test provides a total mark (out of 45) and separate strand marks: a score out of 13 for CN, 8 for NF, 10 for calculation, 4 for shape, 7 for measurement, and 3 for handling data. For the present study, a total numerical score was calculated by combining the CN and NF scores, a total geometrical score was calculated by combing the shape, measurement, and handling data scores, and calculation scores represented arithmetical ability.

### 2.3. Data Analysis

Calculations of descriptive statistics and Spearman’s correlations were performed using IBM SPSS 28.0. To test H2, mediation effects were verified by indirect effects (Williams and MacKinnon 2008) using IBM SPSS Amos 26. Bias-corrected bootstrapped point estimates for the indirect effects of FMS on mathematics achievement were estimated, considering 95% confidence intervals. Significant indirect effects were considered if 95% confidence intervals did not include zero. Bias corrected intervals supported by 5000 bootstrapping samples were used to make inferences. Bootstrapping procedures have been recommended as more efficient and powerful in detecting indirect effects than alternative approaches (Williams and MacKinnon 2008).

## 3. Results

### 3.1. Preliminary Analysis

Of the 155 participants who completed the research tasks, one participant did not complete the mathematics test paper. Their data for the FMS and spatial ability tests were removed. The data for ID and ES spatial ability were also removed. This was because ceiling effects for ID spatial ability (skewness: −1.627) and floor effects for ES spatial ability (skewness: 1.737) were discovered. Participants scored, on average, very highly (over 85%, M = 13.65) on the ID spatial ability test, with 35% of participants scoring the maximum score of 16, whereas participants scored on average very low on the test of ES spatial ability (M = 2.88), with 36% of participants scoring the minimum score of 0. In addition, no extreme outliers were identified on the remaining data (using the Tukey method on SPSS). These preliminary analyses resulted in a final sample size of 154 participants (mean age: 8.04, SD = .302); with 69 males (mean age: 8.05, SD = .315) and 85 females (mean age: 8.03, SD = .293).

### 3.2. Descriptive Statistics and Correlations

On average, the scores for all the different FMS were above 50%. Participants scored, on average, above 50% on the tests of IS and ED spatial ability (M = 13.17, out of 25, and M = 16.40, out of 22, respectively). Participants also scored, on average, 50% or above on most of the mathematics abilities. Performance on shape was the exception (M = 1.43, out of 4).

To test H1 that FMS are positively related to mathematics achievement and to explore which specific FMS are related to which specific mathematics abilities, Spearman correlation coefficients were calculated (see Table 1), as the data were not normally distributed (D_(154)_ > .086, *p* < .007). To summarise, across the whole sample, analyses found that total FMS ability was significantly positively correlated with total mathematics score (r_s_ = .171, *p* = .035). When breaking down FMS into the three components that form it, total locomotion score also significantly positively correlated with total mathematics score (r_s_ = .166, *p* = .040). There were no significant correlations between balance (stability) and mathematics ability, nor total object manipulation score and total mathematics score, however, total object manipulation score significantly positively correlated with measurement score (r_s_ = .190, *p* = .018). Correlation analyses were also conducted between FMS and spatial ability, and mathematics ability and spatial ability. Total FMS score only significantly correlated positively with IS spatial ability for the whole sample (r_s_ = .228, *p* = .004). Total FMS score did not correlate with any other spatial ability. In addition, across the whole sample, all spatial ability skills significantly positively correlated with all mathematics abilities.

### 3.3. Mediation Analysis

**Figure 4:**
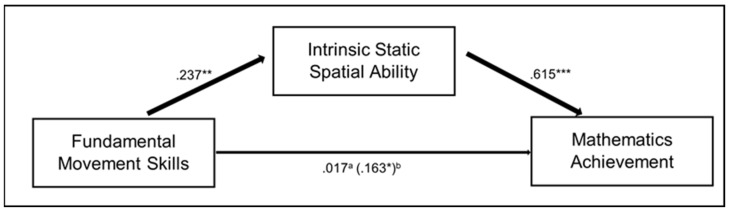
Mediation model showing the effect of FMS on mathematics achievement, as mediated by IS spatial ability. Note: Standardised coefficients reported. * *p* < .05, ** *p* < .01 *** *p* < .001. ^a^ Standardised direct effect coefficient. ^b^ Standardised total effect coefficient.

As a significant positive effect was found between total FMS score and total mathematics score (β = .498, *p* = .043); a mediation analysis of the effect of spatial ability on this relationship was conducted, testing H2. Only IS spatial ability met the assumptions of a regression analysis and was the only spatial ability that had a positive correlation with total FMS score, therefore, a mediation analysis using a 5000-sample bootstrap analysis with 95% confidence intervals was only completed with IS spatial ability. The results show that IS spatial ability fully mediated the relationship between FMS and mathematics ability. Path a (i.e., FMS on IS spatial ability) and path b (i.e., IS spatial ability on mathematics achievement) were both significant (β = .368, *p* = .003 and β = 1.211, *p* < .001, respectively). The direct effect of FMS on mathematics achievement in the presence of IS spatial ability was not significant (β = .053, *p* = .770), however, the indirect effect of FMS on mathematics achievement was significant (β = .445, *p* = .005, 95% CI: [.139 to .804]). This highlights that IS spatial ability fully mediated the relationship between FMS and mathematics achievement, see Figure 4.

## 4. Discussion

The aim of this study was to investigate the relationship between FMS and mathematics achievement, and whether spatial ability mediated this relationship in Year 3 children. Regarding the first hypothesis, the results showed FMS to be significantly positively related to mathematics achievement, supporting H1. However, not all FMS were significantly correlated with all mathematics abilities. In relation to the second hypothesis, IS spatial ability, as assessed by the CEFT, mediated this relationship, providing only partial support for H2.

### 4.1. Relation between FMS and Mathematics Achievement

The main finding of this study confirms that there is a significant, but weak, correlation between FMS and mathematics achievement. This supports previous research that found that motor skills were positively related to mathematics achievement in school aged children. For example, the systematic review by Macdonald et al. (2018) found that motor skills have a weak positive association with achievement in mathematics across school aged children and adolescents. Further, de Bruijn et al. (2019) found that FMS were related to mathematics achievement in children a school year older than those in the current study. This research adds to the literature suggesting that the more developed and mature one’s overall FMS are, the better one’s overall mathematics achievement.

However, this study specifically found a positive correlation between total FMS ability and arithmetical (calculation) and geometrical (measurement) ability, but not numerical ability. No FMS measure correlated with either of the two numeracy skills assessed: counting and knowing number facts. This refutes previous research that found that locomotion and stability skills correlated with basic numeracy skills (de Waal 2019). Further, this study found that object manipulation skills, specifically throwing, were related to geometrical ability, as assessed by understanding measurement, which refutes de Waal (2019) who found no relation between throwing and mathematical abilities. This may be explained by the different types of throwing being assessed. For example, in the current study, children needed to throw one beanbag into each of the five boxes that are placed in increasing distance away from the child, requiring the understanding of changing one’s force in accordance with distance, whereas the children in de Waal’s (2019) study had to only throw a ball over a 7.5 m distance, thus not requiring the calculation of different distances.

The positive relationship found between FMS and mathematics might be explained by cognitive and motor functions using the same brain regions. Research proposes that physical activity and fitness improve cognitive performance by creating neural changes, such as increased cerebral blood flow, upregulation of brain-derived neurotrophic factors, and increase in synaptic plasticity in the brain regions responsible for learning (Best 2010; Etnier et al. 1997). Further, both FMS and cognitive skills involve monitoring, planning, and adaptively changing based on the information provided in the environment, therefore, motor and cognitive skills co-activate the same brain areas—specifically, the prefrontal cortex, cerebellum, and basal ganglia (Diamond 2000). If one has more developed motor skills, this may enhance the connections between these brain areas, improving the development of executive function skills, which may facilitate children’s learning of mathematics, resulting in higher mathematics achievement.

### 4.2. Spatial Ability as a Mediator

Our results are in accordance with previous research that found a positive relationship between all four spatial abilities and arithmetical, numerical, and geometrical ability (Gilligan et al. 2019; Xie et al. 2020). However, only IS spatial ability was positively related to FMS, specifically running, jumping, and total object manipulation scores, which refutes previous research. For example, throwing was positively correlated to performance on the water level test (ES spatial ability) (Jansen and Pietsch 2022), and physical activity and coordinative exercise (such as juggling, which involves object manipulation skills) were positively correlated to ID spatial ability, where individuals score higher on tests of mental rotation (Jansen et al. 2011; Pietsch and Jansen 2012).

The results demonstrated that IS spatial ability mediated the relationship between FMS and mathematics. This highlights that children who had more developed FMS had higher mathematics achievement, and this may be due to them performing better on the CEFT. They were better at finding a shape in a picture by ignoring irrelevant information (disembedding). This could be explained by motor experts having greater visual perception skills. Elite athletes have greater visual perception skills; they are quicker at moving their eyes and detecting valuable perceptual cues whilst ignoring irrelevant information in the environment to perform highly in their sport (Voss et al. 2010). Therefore, the children with mature FMS might engage in more sport and physical activity than children with less developed FMS, which may result in better visual perceptual skills and IS spatial ability, potentially explaining why they score higher in the CEFT and mathematics assessments.

ID and ES spatial ability could not be included in the mediation analysis due to the tests not assessing the child’s true spatial ability, and thus relationships were not found with FMS scores. This study used the same water level test as Jansen and Pietsch (2022) but on a different sample age group. Jansen and Pietsch (2022) used the task on a group of 9-year-old children, who are in Piaget and Inhelder’s (1956) conception of space substage IIIb; they are developmentally able to accurately predict the water level without aid as they understand the principle of invariant horizontality, whereas the children in this study were aged 7–8 years. These children are in stage II and think that the water level will remain constantly parallel to the base of the vessel. Therefore, the test may be unsuitable to assess ES spatial ability in children below 8 years old due to their developmental stage of not being able to understand horizontality; a test of ES spatial ability that does not require this knowledge and is more suitable to the abilities of the sample could have found a relationship between FMS and ES spatial ability. Furthermore, the MRT could have been too easy for the children as the time restriction was removed. This could have led to a lack of variability in the results to find a relationship between FMS and ID spatial ability. If these problems are addressed, then these spatial abilities may also mediate the relationship between FMS and mathematics; thus, our finding that only IS spatial ability mediated the relationship between FMS and mathematics should be viewed with caution.

### 4.3. Limitations

This is the first study to our knowledge that looks at the relationship between FMS and mathematics, and in particular whether spatial abilities mediate this relationship. Despite all three FMS constructs, the four spatial abilities described by Uttal et al. (2013), and arithmetical, numerical, and geometrical abilities being assessed, this research study is not without its limitations. First, as this is a cross-sectional correlational study, directions of causality cannot be inferred. Further, this study used a product-based, rather than process-based, FMS assessment; the quality of the children’s FMS was not explored, which could be a better indication of one’s FMS ability and have stronger relationships to spatial and mathematical skills. Furthermore, two of the spatial tests resulted in ceiling and floor effects—the animal stimuli MRT and the water level test, respectively. Using different tests to assess ID and ES spatial ability may produce more reliable insights into the children’s spatial ability. In addition, logical reasoning was not assessed in this study. Xie et al. (2020) incorporated logical reasoning in their definition of mathematical ability. Future studies should incorporate tests to assess logical reasoning, as solving logical reasoning problems requires the individual to create visual representations and transformations (Oostermeijer et al. 2014), thus tapping into spatial ability. Therefore, logical reasoning could be strongly related to FMS, but this relationship was not investigated in this study. A further limitation of this study is that no control task was included in this correlational study. Future studies should include a control for potential factors that could also cause these variables to correlate, for example, intelligence, as this cognitive process is correlated to mathematical skills in children (Filippetti and Richaud 2017).

### 4.4. Implications

Despite these limitations, the results suggest some future practical implications. Primary school teachers could begin to feel encouraged to read that mathematics achievement could be improved without the need for completing more mathematics. Instead, teachers could ensure PE lessons are delivered to improve the children’s FMS, resulting in not only better physical and mental health, but potentially better mathematics achievement in children. However, further research must be completed to ensure that the effects of FMS on mathematics via the development of spatial skills are true, robust, and replicable before changes within the curriculum are made prematurely.

## 5. Conclusions

This study investigated the relationships between FMS, spatial abilities, and mathematics achievement. It found that total FMS was positively related to total mathematics achievement, albeit weakly, and that IS spatial ability mediated this relationship. However, these relationships found should be regarded with caution. Replication of this study is needed to confirm and strengthen the relationships found and whether the implementation of different spatial tests identify different findings. This will provide more conclusive findings on the relationships between FMS and mathematics, and how one may affect the other, and the impact spatial ability has on this relationship.

## Figures and Tables

**Table 1:** Means, standard deviations, and Spearman Rho Correlations Between Fundamental Movement Skills, Spatial Abilities, and Mathematical Abilities for the Whole Sample (*N* = 154).

Variable (Score)	Mean (SD)	1	2	3	4	5	6	7	8	9	10	11	12	13	14	15	16	17	18	19
1. Running	6.45 (.871)	-																		
2. Jumping	3.14 (.943)	.097	-																	
3. Hopping	3.13 (.853)	.199 *	.406 **	-																
4. Total Throw	7.71 (1.256)	.291 **	−.008	.093	-															
5. Kicking	2.47 (.785)	.201 *	−.032	.010	−.004	-														
6. Balance (Stability)	2.91 (.986)	−.024	.157	.296 **	−.091	−.024	-													
7. Total Locomotion	12.72 (1.866)	.551 **	.752 **	.763 **	.156	.063	.224 **	-												
8. Total Object Manipulation	10.19 (1.459)	.339 **	−.046	.060	.831 **	.527 **	−.106	.136	-											
9. Total FMS	25.82 (2.852)	.526 **	.534 **	.665 **	.489 **	.265 **	.459 **	.818 **	.534 **	-										
10. IS Spatial Ability	13.17 (4.419)	.183 *	.172 *	−.007	.136	.138	.120	.144	.175 *	.228 **	-									
11. ED Spatial Ability	16.40 (3.928)	−.029	−.031	−.062	.167 *	−.007	−.005	−.073	.123	.007	.462 **	-								
12. Counting	9.29 (2.373)	.181 *	.091	.048	.120	.102	−.031	.119	.060	.156	.587 **	.445 **	-							
13. Number Facts	5.47 (1.900)	.144	.049	.094	.041	.025	−.023	.099	.026	.085	.504 **	.406 **	.694 **	-						
14. Calculation	5.90 (2.370)	.244 **	.190 *	.120	.066	.034	−.009	.230 **	.060	.194 *	.587 **	.382 **	.758 **	.703 **	-					
15. Shape	1.43 (1.034)	.132	.115	.087	.078	−.014	−.056	.128	.039	.108	.366 **	.303 **	.509 **	.422 **	.536 **	-				
16. Measurement	3.95 (1.911)	.276 **	.019	.069	.204 *	.062	−.029	.135	.190 *	.191*	.533 **	.328 **	.676 **	.588 **	.642 **	.412 **	-			
17. Handling Data	1.99 (1.029)	.009	.025	.068	.001	.001	−.049	.032	−.017	−.013	.346 **	.283 **	.499 **	.398 **	.493 **	.380 **	.391 **	-		
18. Numerical Ability	14.76 (3.956)	.183 *	.086	.076	.084	.069	−.032	.126	.095	.136	.595 **	.469 **	.937 **	.896 **	.797 **	.513 **	.692 **	.489 **	-	
19. Geometrical Ability	7.37 (3.121)	.207 *	.066	.093	.144	.049	−.049	.136	.126	.153	.564 **	.401 **	.749 **	.636 **	.737 **	.706 **	.868 **	.693 **	.759 **	-
20. Total Maths Score	28.03 (8.699)	.226 **	.110	.098	.110	.067	−.035	.166 *	.111	.171 *	.630 **	.458 **	.897 **	.823 **	.905 **	.632 **	.799 **	.603 **	.939 **	.904 **

Note: * *p* < .05, ** *p* < .01.

## Data Availability

The data are not publicly available due to ethical restrictions.
